# Preventive *Trichuris suis* ova (TSO) treatment protects immunocompetent rabbits from DSS colitis but may be detrimental under conditions of immunosuppression

**DOI:** 10.1038/s41598-017-16287-4

**Published:** 2017-11-28

**Authors:** Irina Leonardi, Alexandra Gerstgrasser, Thomas S. B. Schmidt, Flora Nicholls, Bernhard Tewes, Roland Greinwald, Christian von Mering, Gerhard Rogler, Isabelle Frey-Wagner

**Affiliations:** 1 Division of Gastroenterology and Hepatology USZ, Zurich, Switzerland; 2 Institute of Molecular Life Sciences, University of Zurich, Zurich, Switzerland; 3 Central Biological Laboratory, USZ, Zurich, Switzerland; 4 Research and Development, Dr. Falk Pharma GmbH, Freiburg, Germany; 5 Present Address: Gastroenterology and Hepatology Division, Joan and Sanford I. Weill Department of Medicine, Weill Cornell Medicine, New York, USA

## Abstract

*Trichuris suis* ova (TSO) have been tested for therapeutic application in inflammatory bowel diseases (IBD) yet understanding of the underlying mechanisms and safety in an immunocompromised host is limited due to lack of a suitable animal model. We used a recently established rabbit model of dextran sodium sulphate (DSS) induced colitis to study the efficacy, mechanisms and safety of TSO therapy in immunocompetent and immunosuppressed animals. TSO treatment prevented the DSS induced weight loss, delayed the onset of DSS induced symptoms by 2 days and significantly reduced the disease activity (DAI). TSO treatment protected caecal histology and prevented the colitis-associated loss in faecal microbiota diversity. Mainly the transcriptome of lamina propria mononuclear cells (LPMC) was affected by TSO treatment, showing dampened innate and adaptive inflammatory responses. The protective effect of TSO was lost in immunosuppressed rabbits, where TSO exacerbated colitis. Our data show that preventive TSO treatment ameliorates colitis severity in immunocompetent rabbits, modulates LPMC immune responses and reduces faecal dysbiosis. In contrast, the same TSO treatment exacerbates colitis in immunosuppressed animals. Our data provide further evidence for a therapeutic effect of TSO in IBD, yet caution is required with regard to TSO treatment in immunosuppressed patients.

## Introduction

The aetiology of inflammatory bowel disease (IBD) is complex and not fully understood, yet. Nonetheless, the increasing incidence in developing nations suggests that the environment plays a critical role in the pathogenesis of both ulcerative colitis (UC) and Crohn’s disease (CD)^1^. Among various factors, a clear inverse correlation exists between the prevalence of IBD and soil-transmitted helminthic infections^2^. According to the old-friends hypothesis, helminths are intestinal symbionts that co-evolved with the adaptive immune system and are thereby essential for its proper maturation and functioning^3^.

Immune-modulation exerted by helminths has been proposed for the prevention and treatment of various immune-related diseases^4^. Different nematode species have been shown to suppress inflammation in mouse models of experimental colitis, mainly through the action of their excretory secretory (ES) products^5^. ES products of *Ancylostoma species* reduced Th1 and Th17 responses and ameliorated inflammation in models of chemically induced colitis^6–8^. *Acanthocheilonema vitae* secreted a Cystatin that reduced inflammatory macrophages in the colon and the local production of inflammatory cytokines while increasing the numbers of regulatory T cells (Tregs) in dextran sodium sulphate (DSS) colitis^9^. ES produced by *Nippostrongylus brasiliensis* directly suppressed IL-12p40 production by DCs^10^ *. Heligmosomoides polygyrus* caused the expansion of tolerogenic DCs that blocked antigen-specific IFNγ and IL-17 T cell responses and attenuated colitis^11,12^. Mice previously infected with *Trichuris muris* recovered faster from DSS colitis and showed an increased regeneration of the mucosa suggesting that the protective effect of intestinal nematodes can persist after their clearance from the intestine^13^.

Besides their immunomodulatory role, intestinal nematodes might positively affect the dysbiosis observed during intestinal inflammation. The microbiota of IBD patients is characterized by decreased diversity, reduced abundance of *Firmicutes* (CD) or *Bacteroidetes* (UC) and increased abundance of Proteobacteria and/or Actinobacteria^14^. *H. polygyrus* has been shown to increase the abundance of *Lactobacillaceae* in the ileum of infected mice^15,16^. Similarly, the swine parasite *Trichuris suis* affected the abundance of Proteobacteria and Deferribacteres in the pig colon^17^. Whether the modulation of the gut microbiota also contributes to the therapeutic effect of helminths in intestinal inflammation has not been studied so far.

*T. suis* is the main helminth species tested in human subjects. Trials in human patients have been performed by administration of different doses of embryonated *T. suis* ova (TSO)^18^. The current view is that *T. suis* larvae hatch and transiently colonize the human intestine for some weeks without reaching sexual maturity^19^.

A recent Cochrane systematic review came to the conclusion that the evidence in support of the efficacy and safety of TSO for the treatment of IBD remains inconclusive^20^. The initial open-label studies showed promising clinical efficacy and safety^21^. Yet, larger multicentre studies failed to show a significant effect of TSO in comparison to placebo in mild to moderate IBD patients^22^.

The safety of helminth therapy in immunosuppressed individuals, that constitute the majority of IBD patients, is a major concern. Although the initial studies did not report severe adverse effects among the immunosuppressed patients receiving TSO^23^, Kradin *et al*. reported the case of an invasive iatrogenic infection in an immunosuppressed CD patient^24^. Of note, the nature of IBD itself might mask the presence of TSO induced symptoms, thereby complicating the assessment of the side effects of TSO.

To date, the lack of a suitable animal model has hampered detailed investigations into the mechanisms and safety of TSO treatment for IBD. Since the course of a *T. suis* infection in the rabbit intestine is similar to the transient colonisation proposed for humans, we recently developed a colitis model in rabbits by administration of DSS^25^. Here, we used this model to investigate the efficacy and safety of a preventive TSO therapy as proposed for IBD patients for maintenance of remission, in immunocompetent and immunosuppressed animals.

## Results

### TSO prevent colitis in immunocompetent rabbits

**Figure 1:**
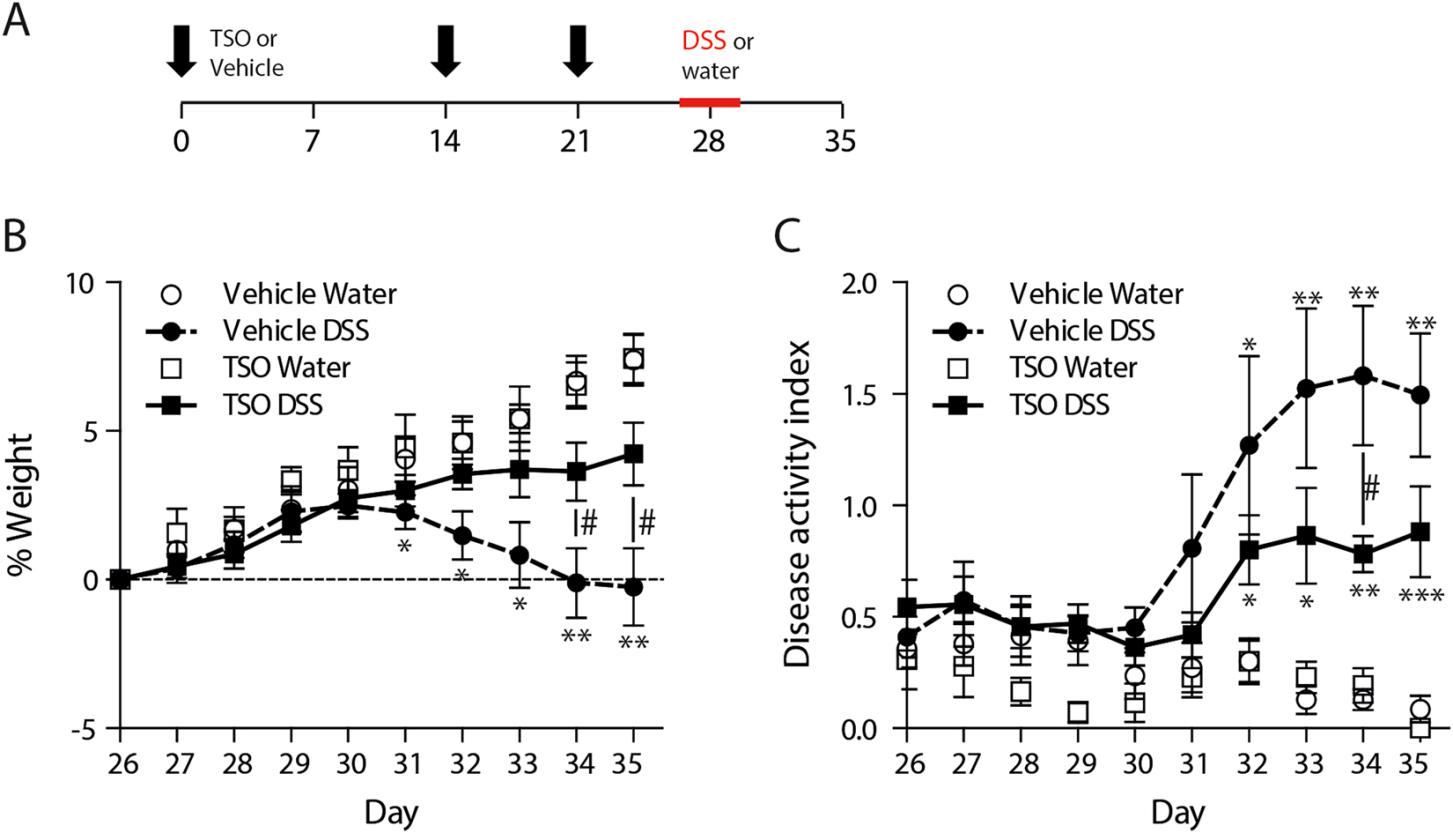
Preventive TSO treatment ameliorates DSS colitis. (**A**) NZW rabbits received 2500 TSO at day 1, 14 and 21 (intra-gastrically). At day 26 colitis was induced by administration of 0.1% DSS in the daily beverage for 5 days. (**B**) Body weight is shown as percentage of individual weight at start of DSS colitis induction (day 26). (**C**) Clinical symptoms (reduction in food and beverage intake, fur cleanliness, weight loss and stool consistency), were scored daily and summarized as disease activity index (DAI). Bars show mean ± SEM, *P < 0.05, **P < 0.01, ***P < 0.001, two-sided p-Value, Mann-Whitney test relative to the vehicle DSS group (#) and to the vehicle water group (*). Data are pooled from two independent experiments with n = 3–4 rabbits per group.

To investigate whether TSO can ameliorate the outcome of colitis, we administered three doses of 2500 TSO prior to the administration of DSS (Fig. 1A). Following DSS administration, vehicle treated rabbits (Veh) rabbits exhibited watery diarrhea and significant weight loss in comparison to the control starting on day five of DSS administration and continuing until the end of the experiment. In contrast, TSO treated animals maintained the constant weight gain observed in age-matched healthy controls (Fig. 1B, Supplementary Figure 1) thus that there was a significant difference in weight change between the TSO DSS and the DSS group on day 9 and 10 after start of DSS administration. Concurrent to weight loss, Veh animals developed severe symptoms. At day 9 after the start of DSS administration, they had reached an average disease activity index (DAI, Fig. 1B) of 1.6 ± 0.8. In contrast, TSO treatment delayed the onset of symptoms and reduced the disease severity significantly (DAI at day 9: 0.7 ± 0.2, P < 0.05 in comparison to the Veh-DSS group). Importantly, TSO treatment alone did not induce any weight loss and no symptoms were observed in healthy animals (Fig. 1B and C).

### TSO prevent severe histopathology in the caecal mucosa

**Figure 2:**
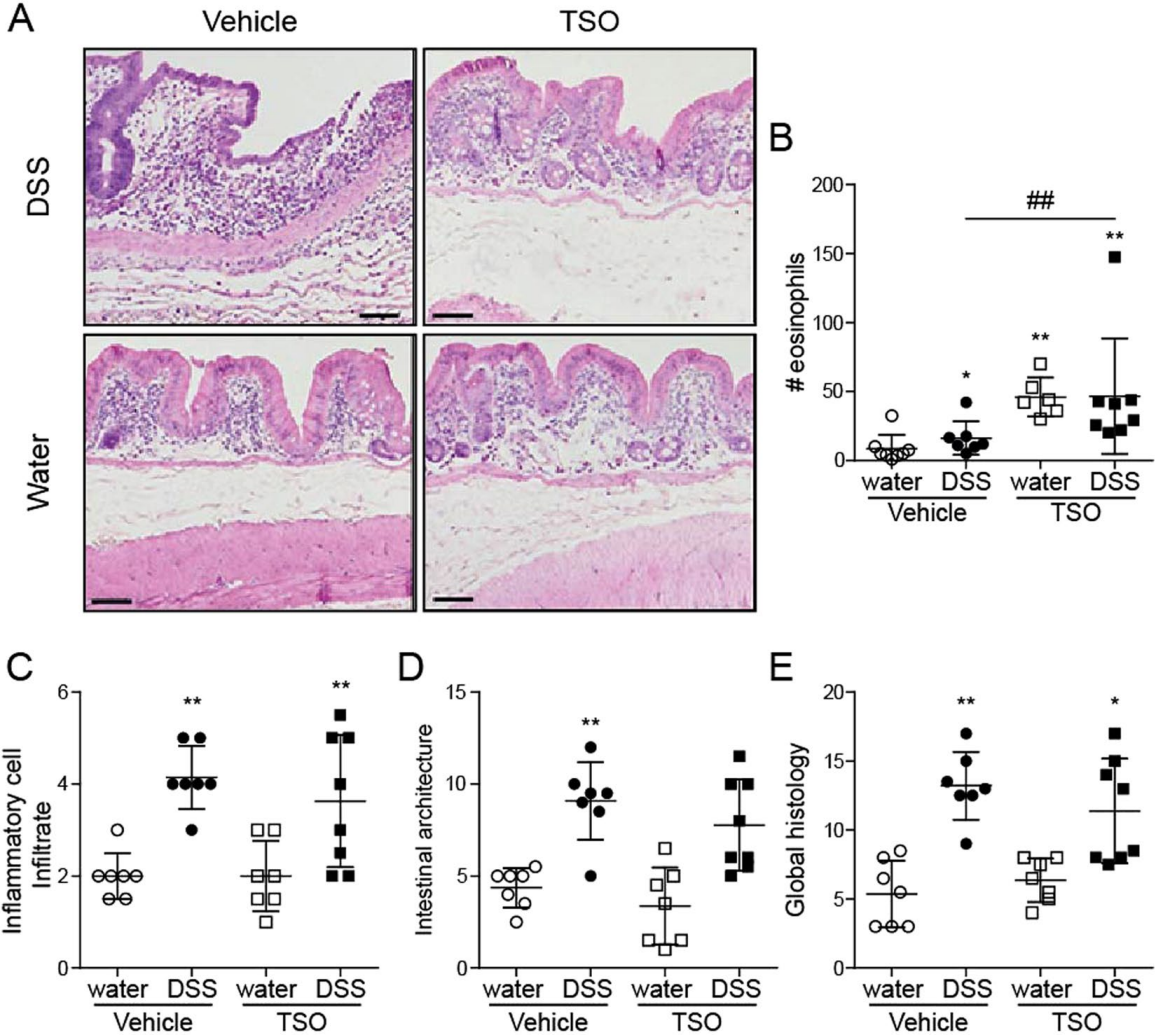
TSO treatment reduces the caecum histopathology of DSS colitis. Caecal samples were collected after euthanasia at day 35. (**A**) HE stained caecum sections from vehicle or TSO treated rabbits with or without colitis induction. Bars = 100 µm. (**B**) Inflammatory cell infiltrate score based on the lymphocyte infiltrate into the lamina propria and epithelium (2–8). (**C**) Intestinal architecture score based on independent scoring of villous stunting, epithelium damage and crypt distortion (3–12). (**D**) Inflammatory cell infiltrate and intestinal architecture score were summarized to a combined histology score (5–20). (**E**) Quantification of the average number of eosinophils within a 40 x field of caecum. Data are pooled from two independent experiments with n = 3–5 animals per group. For the scoring, three caecal samples per rabbit were collected and evaluated separately for at least three rabbits per group. *P < 0.1, **P < 0.05, two-sided p-Value, Mann-Whitney test relative to vehicle water control (*) or to vehicle DSS (#). Dots represent single animals, bars represent mean ± SEM.

**Figure 3:**
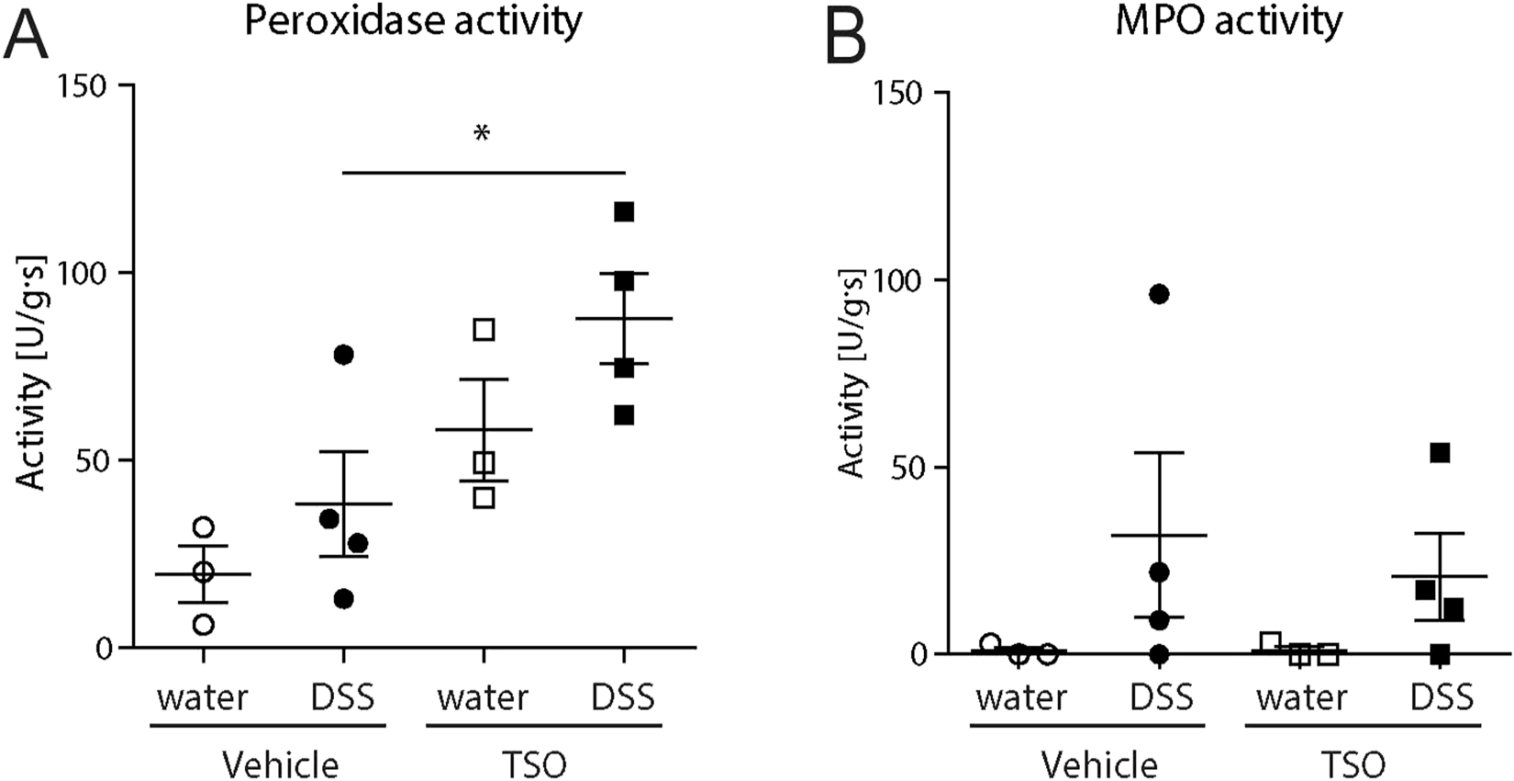
Neutrophil and eosinophil infiltration into the caecum. Infiltration was determined indirectly by measuring the caecal peroxidase activity. Caecal specimens were excised and homogenized. The supernatants were assayed for the determination of peroxidase activity with (**B**) or without (**A**) the selective eosinophil-peroxidase inhibitor aminotriazole (AMT). Activity was normalized to the total protein content as determined by BCA test. Dots represent single animals, n = 3–5 animals per group, bars show mean ± SEM, *P < 0.05, unpaired t test.

We next examined the effect of TSO on the DSS induced caecum inflammation. Histologically, the caecum of TSO treated healthy rabbits did not show any sign of epithelial damage or villous stunting yet some changes in crypt architecture were visible (Fig. 2A). As expected, TSO induced a strong infiltration of eosinophils into the lamina propria even in the absence of DSS administration (Fig. 2B), leading to a significantly increased peroxidase activity (Fig. 3A). In Veh animals, DSS triggered a caecal pathology characterized by profound morphological changes and a marked infiltration of lymphocytes into the lamina propria and into the epithelium (Fig. 2C,D and E). TSO partially protected the caecal mucosa from the DSS-induced morphological changes and reduced lymphocyte infiltration into the mucosa (Fig. 2A). Blinded semi-quantitative scoring of morphological and inflammatory histological parameters showed that TSO prevented the development of histological damage in half of the treated animals (Fig. 2C,D and E). This observation, suggests the existence of individual-specific factors that might modulate the efficacy of TSO treatment. Innate leukocyte invasion was assessed by measurement of the peroxidase activity. The overall peroxidase activity was increased in the TSO treated animals even in the absence of DSS induced injury (Fig. 3A). This increase was due to a TSO induced infiltration of eosinophils as treatment with the eosinophils’ peroxidase (EPO) inhibitor aminotriazole (AMT) reduced the peroxidase activity in the TSO treated animals and revealed a trend towards decreased neutrophil infiltration in the TSO DSS animals (Fig. 3B).

### TSO influence the faecal and caecal microbiota

The immune-effect of intestinal helminths has been linked to the modulation of intestinal microbiota^16^ Since *T. suis* resides in close contact with caecal commensal bacteria, we hypothesized that TSO administration might be associated with distinct changes in the bacterial community in the caecum and, as a consequence, in the faeces. We therefore assessed how *T. suis* influences the microbial community in healthy and colitic rabbits.

**Figure 4:**
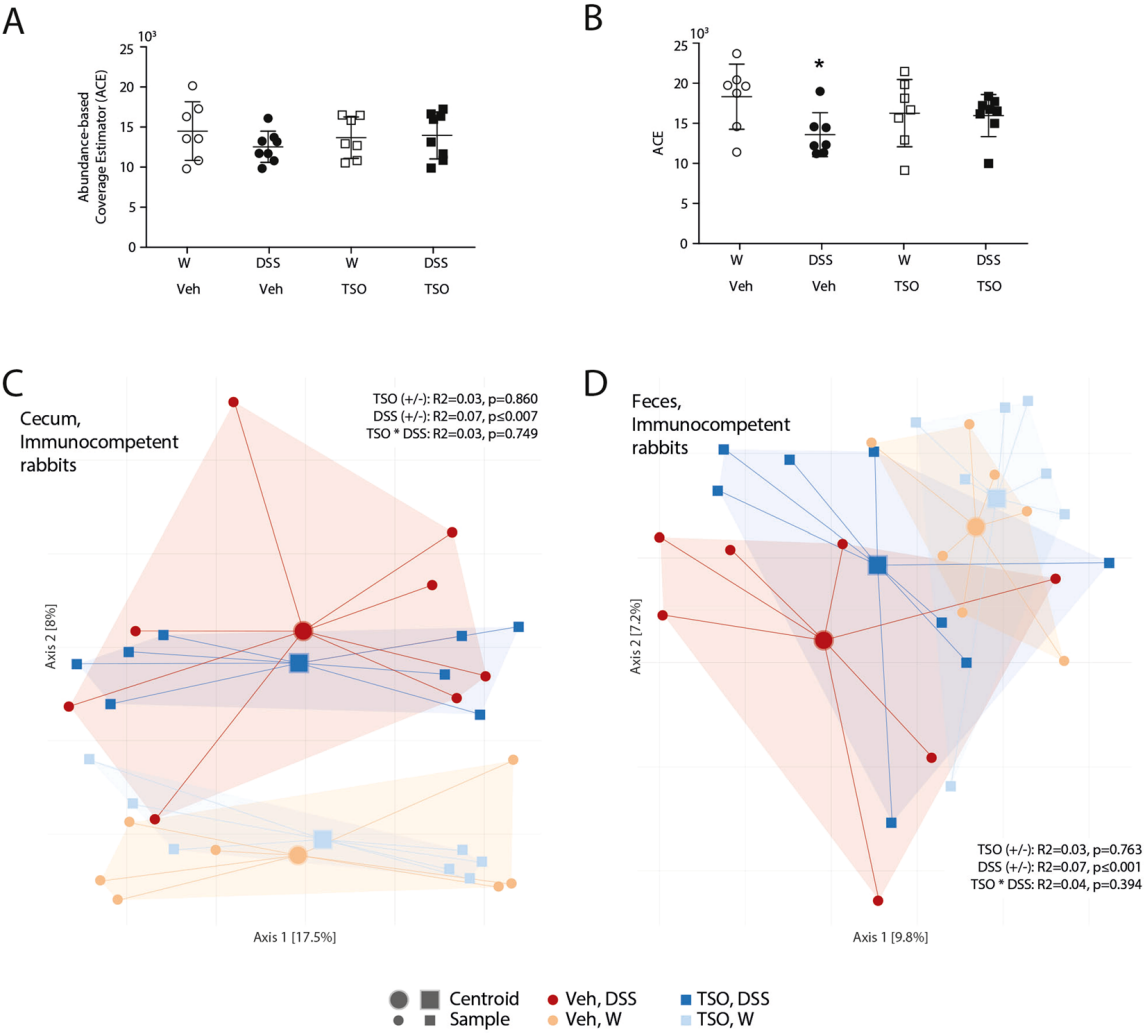
TSO partially prevents the DSS induced shift in gut microbiota composition. Microbiota richness in caecum (**A**) and faeces (**B**) as assessed by abundance based coverage estimator, (ACE). PCoA ordination based on Bray-Curtis dissimilarities show shifts in community composition for caecal (**C**) and faecal samples (**D**) in response to DSS treatment in immunocompetent animals. The percentage of variation explained is given in parentheses, Increasing distance in two-dimensional space represents increasingly dissimilar communities.*P < 0.05, one-sided p-Value, Mann-Whitney test relative to vehicle water control. Dots represent single animals n = 7–8 animals per group, bars represent mean ± SEM.

In faeces, DSS treatment caused a statistically significant decrease in both richness (P < 0.05, Abundance-based Coverage Estimator, ACE) and Shannon diversity^26,27^. This loss of richness and diversity was significantly reduced by pre-treatment with TSO (Fig. 4A,B).

**Figure 5:**
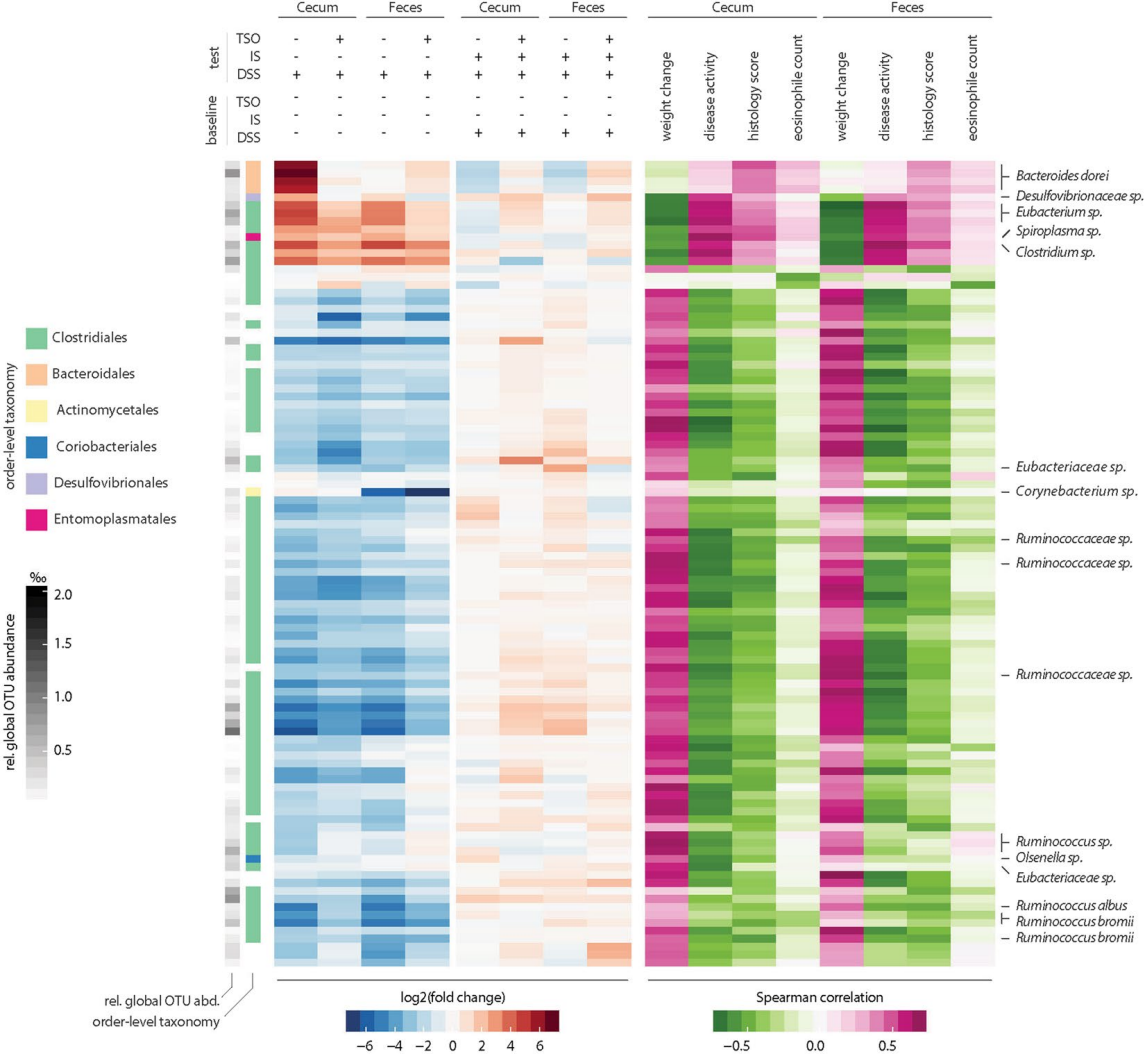
Association of individual OTUs with treatment and colitis severity. Differential OTU abundance in the caecal microbiota between treatment and baseline conditions were assessed using the R package *edgeR*. The top 100 OTUs showing the most significant abundance shifts (FDR < 0.01) in at least one tested condition or a strong correlation to at least one parameter of colitis severity (Spearman correlation, rho <|0.5|) are shown. The leftmost columns indicate global OTU size (relative abundance in the entire dataset) in shades of grey and order-level OTU taxonomy in pastel colours (white indicates unknown taxonomy at order level). Blue/red heat maps on the left indicate the fold change (log2|FC|) of OTU abundances between the test conditions; pink/green heat maps on the right indicate correlation strength to colitis severity parameters. OTUs were clustered on the y-axis based on Pearson distances of their fold changes across conditions.

We next compared the groups in terms of community composition in a Principal Coordinate Analysis (PCoA) based on Bray-Curtis dissimilarities. Permutation multivariate ANOVA (PERMANOVA) analysis revealed a significant effect of DSS on both caecal and faecal communities (P < 0.05, Fig. 4C,D). Although global analysis did not reveal clear clustering by TSO treatment, the abundance of several bacterial taxa showed significant variation between the groups. In faeces, TSO prevented the DSS-induced increase in members of the phylum *Bacteroidetes* and the decrease in *Firmicutes*. In the caecum, similar trends, although not significant, were observed (Supplementary Figure 2). To identify colitis-associated bacteria and investigate how TSO influenced their abundance, EdgeR was used to identify the differentially abundant OTUs with respect to the untreated control group (Fig. 5). Following DSS*, Bacteroidetes dorei, Desulfovibrionaceae sp, Eubacterium sp, Spiroplasma sp* and *Clostridium sp* abundances increased significantly relative to water-treated controls (FDR < 0.01). Further, the relative abundances of these OTUs in both caecum and faeces correlated strongly with different indicators of colitis severity (weight change, disease activity and histology score). In contrast, the frequency of several *Ruminococcaceae sp*. OTUs and other *Clostridiales* decreased upon DSS and had a strong negative correlation with colitis severity. Further, TSO prevented the colitis-associated peak of *B. dorei* and *Desulfovibrionaceae sp*. in the caecum.

### TSO modulate the IEC and LPMC transcriptome

To gain further insights into the mechanisms of TSO treatment, we performed genome wide expression analysis on intestinal epithelial cells (IEC) and lamina propria mononuclear cells (LPMC) isolated from caecum samples by RNA sequencing (RNAseq). Hierarchical clustering (Supplementary Figure 3) revealed distinct expression profiles for EC and LPMC and highlighted a strong effect of TSO treatment particularly in LPMC, whereas the effect in EC was milder.

**Figure 6:**
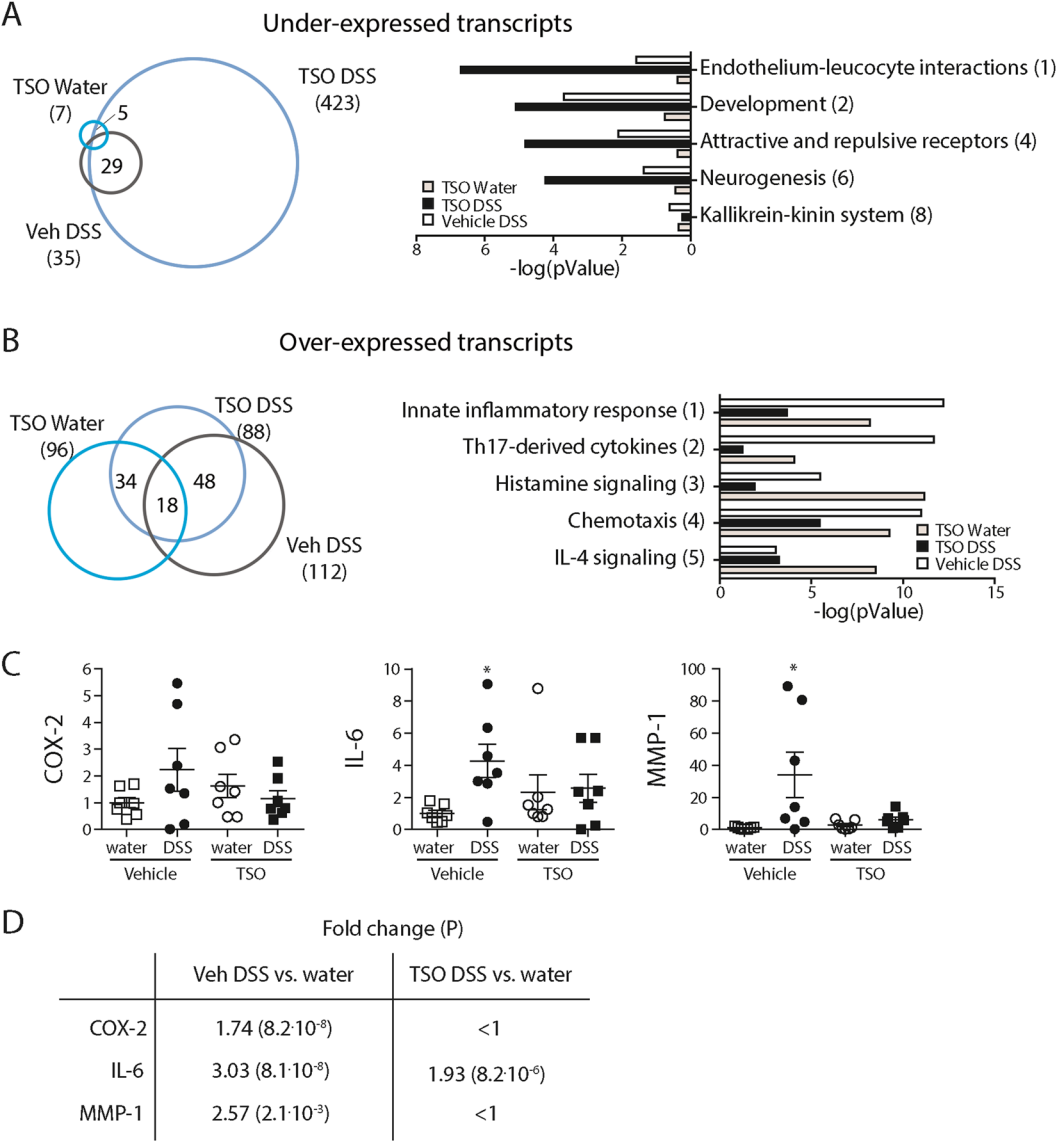
Differential mRNA expression in LPMC. Genome wide expression analysis by RNAseq was performed with RNA from LPMCs isolated from rabbit caeca (n = three per group). Expression profiles of the different treatment groups were sorted by hierarchical clustering. Left) Number of under- (**A**) and over-expressed (**B**) mRNAs in the TSO DSS, Veh DSS and TSO Water groups in comparison to the control Veh Water group. Transcripts having a log2FC > |1| and P < 0.05 were considered as differentially expressed and included in further analyses. Right) Significantly affected Process Networks (PN) as identified by MetaCore™. PN without relevance to the investigated tissue and model were omitted. Numbers in parentheses provide the PN rank. (**C**) Validation of differential mRNA expression of COX-2, IL-6, and MMP-1 in LPMC (data pooled from two separate experiments, with n = 3–4 per group) by rt-qPCR. Expression was calculated with the ΔΔCt method relative to GAPDH and gave similar results as RNAseq (**D**). *P < 0.05, two-sided p-Value, Mann-Whitney test relative to vehicle water control if not indicated otherwise. Dots represent single animals, bars represent mean ± SEM.

In LPMC, we identified 511 differentially expressed genes (log2 fold change |FC| > 1, FDR corrected p-Value < 0.05) in the TSO DSS animals in comparison to the Veh Control group. 423 genes were expressed at lower level and 88 were up-regulated in the TSO DSS animals (Fig. 6).

Genes with significantly altered mRNA levels were analysed by MetaCore to determine the biological relevance of their differential expression. Enrichment analysis showed that in the healthy mucosa, TSO influenced the expression of genes known to be associated with parasitic infections such as TCR signalling, phagocytosis, innate inflammatory response and macrophage migration inhibitory factor (MIF) signalling. Interestingly, 42% of the genes over-expressed in response to TSO in IEC and LPMC have been shown to be over-expressed in the caecum of *T. muris* infected mice^28^ (Supplementary Figure 4 and Supplementary Table 1). In the LPMC of DSS treated animals, TSO treatment led to the down-regulation of genes involved in cell-adhesion (Fig. 6A) and limited the expression of genes involved in innate inflammation and Th17 pathways (Fig. 6B). Differential expression of selected transcripts was verified by rt-qPCR and revealed a similar fold change as obtained by mRNA sequencing (Fig. 6C,D).

### TSO exacerbate colitis in immunosuppressed animals

**Figure 7:**
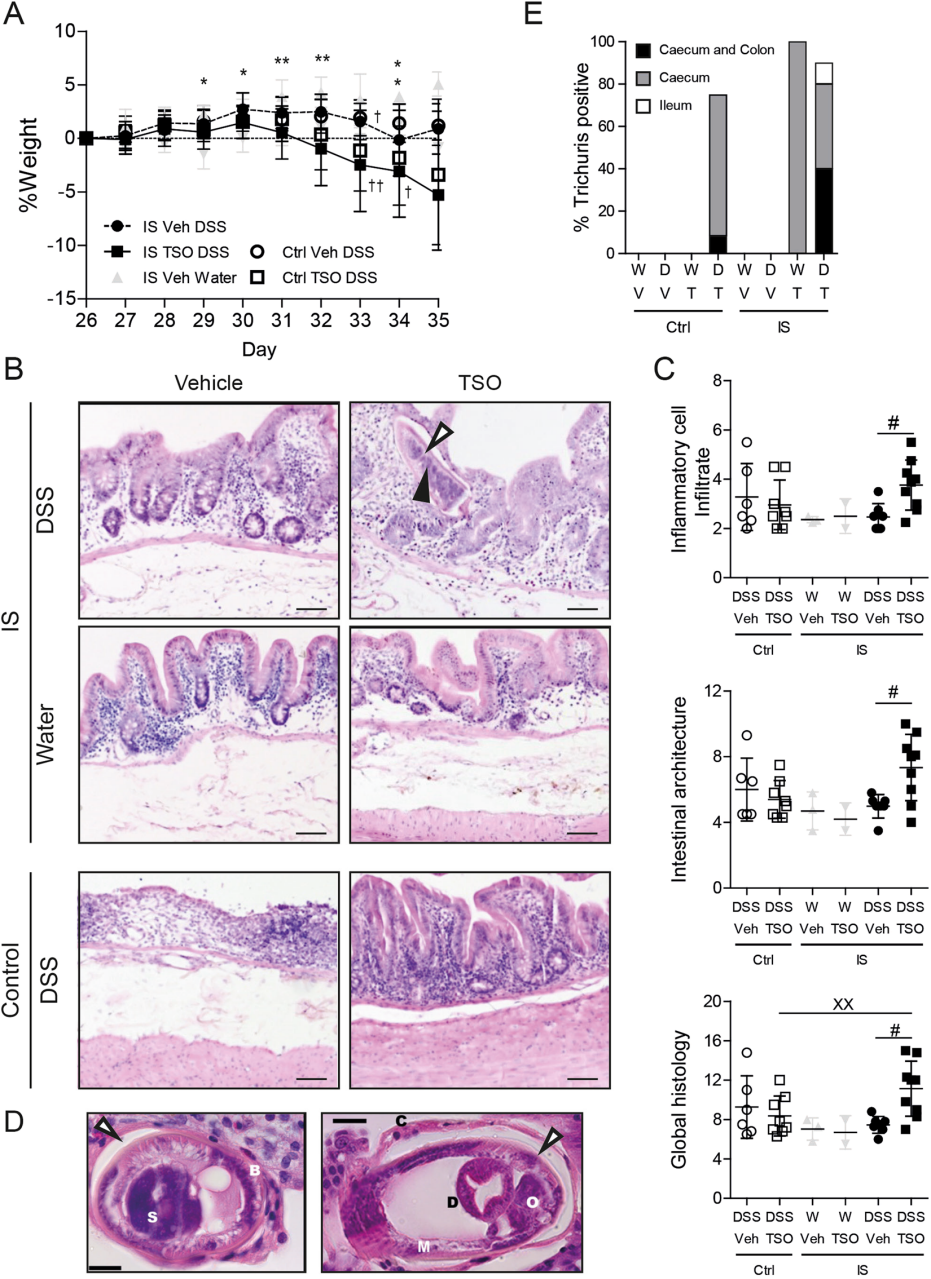
Immunosuppression abrogates the protective effects of TSO and exacerbates the DSS induced damage and inflammation of the caecal mucosa. (**A**) Body weight change relative to the baseline at DSS colitis induction (day 26). (**B**) Representative HE stained specimens of caecal mucosa (day 35). IS: immunosuppressed rabbits, control: immunocompetent rabbits. Bars = 100 µm. 3 specimens per rabbit were assessed. Filled arrowhead: *T. suis* schistosome. (**C**) Inflammatory cell infiltrate score based on the lymphocyte infiltrate into the lamina propria and epithelium (2–8). Intestinal architecture score based on independent scoring of villous stunting, epithelium damage and crypt distortion (3–12). Inflammatory cell infiltrate and intestinal architecture score were summarized to a combined histology score (5–20). (**D**) HE stained section of the caecal mucosa of IS TSO-DSS rabbits showing the presence of adult *T. suis*. Left panel: longitudinal section of the posterior part; Right panel: cross section of the posterior end of the parasite. S: stychosome nucleus; Bb: bacillary band; C: cuticle; O: reproductive organs; D: digestive tract; M: muscle layer. Bars = 20 µm. Empty arrowhead: syncytial tunnel derived from the host’s caecal epithelium. (**E**) Percentage of animals tested positive for *Trichuris* by PCR in different regions of the gastrointestinal tract (caecum, colon and ileum). W: water, D: DSS, V: Vehicle, T: TSO, Ctrl: Control, IS: immunosuppressed. Dots represent single animals, bars represent mean ± SEM, data were pooled from three independent experiments, n = 2–4 animals per group. *P < 0.05, **P < .01, two-sided p-Value, Mann-Whitney test between IS Veh DSS (n = 8^‡^) and IS TSO DSS (n = 9^‡^). ^‡^Number of animals at the start of the experiment. 3 IS TSO DSS and 1 IS Veh DSS animals reached the euthanasia criteria and were sacrificed at the indicated time point (†).

Since most IBD patients are on immunosuppressive therapy we investigated how TSO treatment affects DSS colitis in immunosuppressed (IS) rabbits (Supplementary Figure 5A). Cyclosporine and Methylprednisolone are used in IBD to treat severe acute UC either as monotherapy or in combination. The efficiency of immunosuppression by Cyclosporine and Methylprednisolone was confirmed by differential blood count (Supplementary Figure 5C). Of note, a reduced weight gain was observed in IS animals independently of TSO treatment (Supplementary Figure 5B). After 2 weeks of IS treatment leukocyte count fell below 5.10^9^ cells/µl, with lymphocytes being the most affected cell population. In IS animals, TSO did not induce any clinical symptom and no adverse effects were observed in the absence of colitis. In contrast, colitis induction was followed by severe weight loss in the TSO treated IS rabbits (Fig. 7A). Further, TSO exacerbated the DSS induced symptoms that three out of nine IS animals had to be euthanized prior to the scheduled end of the experiment.

Histological examination (Fig. 7B), showed increased villous stunting and altered crypt architecture in IS TSO DSS animals as well as enhanced infiltration of lymphocytes into the epithelial layer. The extent of immune cell infiltration and morphological alteration were elevated in comparison to the IS DSS control group (Fig. 7C). Further examination revealed the presence of TSO larvae in the caecal mucosa of 5 out of 9 IS TSO DSS rabbits. Parasite size and morphological features were characteristic of a late larval or early adult stage, with distinguishable tripartite oesophagus and well-developed reproductive organs (Fig. 7D). No un-embryonated eggs were detected by faecal sedimentation-flotation, suggesting that *T. suis* had not reached sexual maturity (data not shown). Larvae were not observed in other parts of the intestine (ileum, proximal and distal colon). Yet, PCR-detection revealed the presence of *T. suis* in the colon of 4 out of 10 IS TSO DSS and 2 out of 2 IS TSO Water rabbits (Fig. 7E). The spread of *T. suis* outside the caecal niche confirms that IS rabbits fail to control the helminth infection. Analysis of the peroxidase activity in IS animals, only, showed a small increase after TSO treatment for both the global peroxidase activity and the MPO specific activity (Supplementary Figure 6). This was in accordance with the absence of a significant increase in eosinophilic infiltrate observed by histology (data not shown).

### Immunosuppression increases the frequency of colitis-associated bacteria

**Figure 8:**
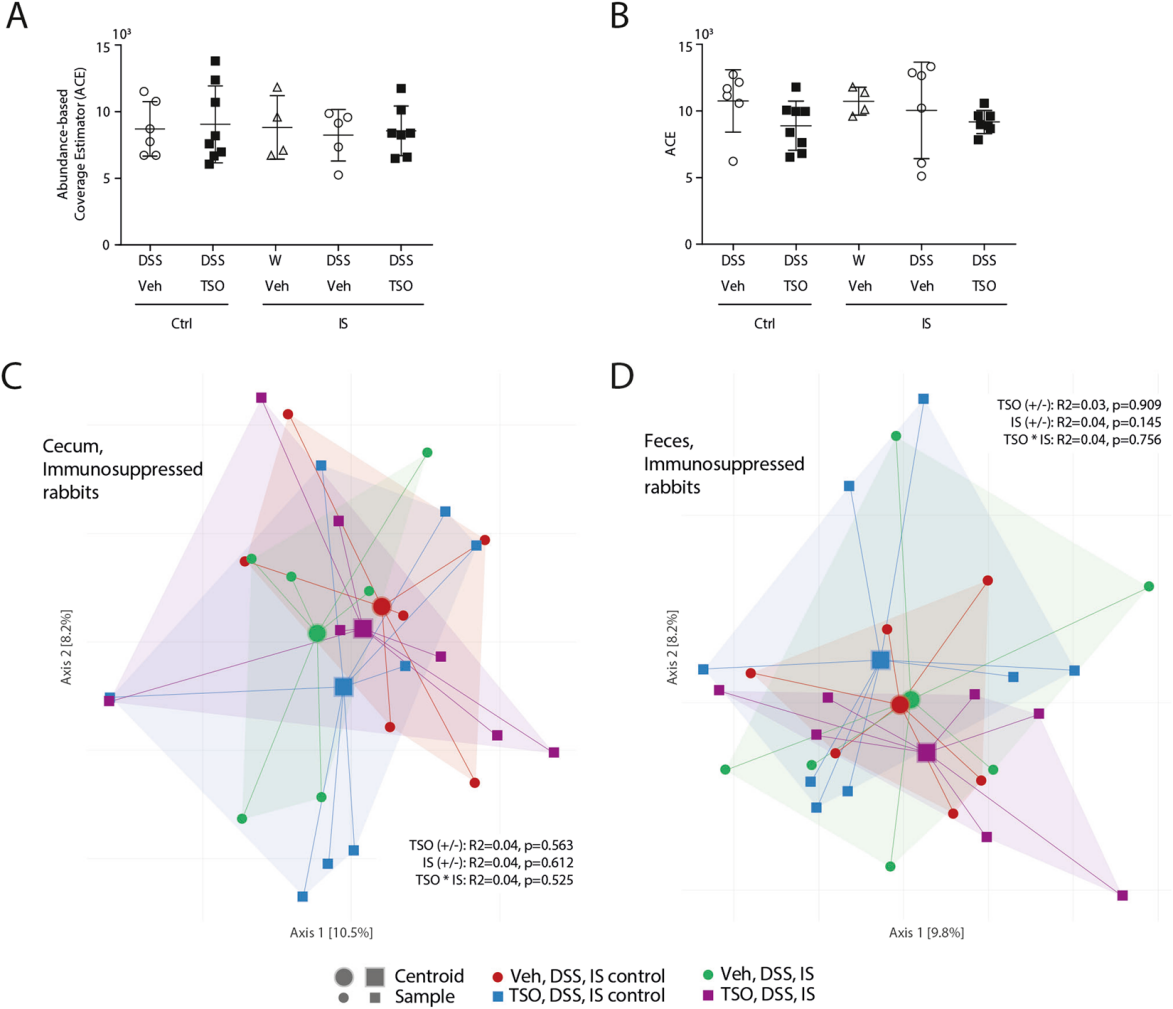
TSO do not affect gut microbiota composition in immunosuppressed animals. Microbiota richness in caecum (**A**) and faeces (**B**) as assessed by ACE. PCoA ordination based on Bray-Curtis dissimilarities show no shifts in community composition for caecal (**C**) and faecal samples (**D**) in immunosuppressed animals. The percentage of variation explained is given in parentheses; increasing distance in two-dimensional space represents increasingly dissimilar communities. Dots represent single animals, n = 4–8 animals per group. Bars represent mean ± SEM.

We next assessed whether the abrogation of the protective effect of TSO observed in IS rabbits was accompanied by shifts in the intestinal bacterial community. In IS rabbits, TSO treatment did not affect the community evenness, richness and between-sample β-diversity as assessed by Bray-Curtis-dissimilarity (Fig. 8A and B).

At OTU level, several strains associated with a more severe colitis severity were decreased in the DSS IS animals in comparison to immunocompetent animals, which correlates with the observed amelioration of colitis severity (Fig. 5). In contrast, concomitant IS and TSO treatment caused an increase of colitis associated OTUs. In particular, TSO led to an increased frequency of *B. dorei*, *Desulfovibrionaceae sp* and *Eubacterium sp* OTUs in immunosuppressed animals.

### TSO decreases the expression of alternative macrophage activation markers in immunosuppressed rabbits

**Figure 9:**
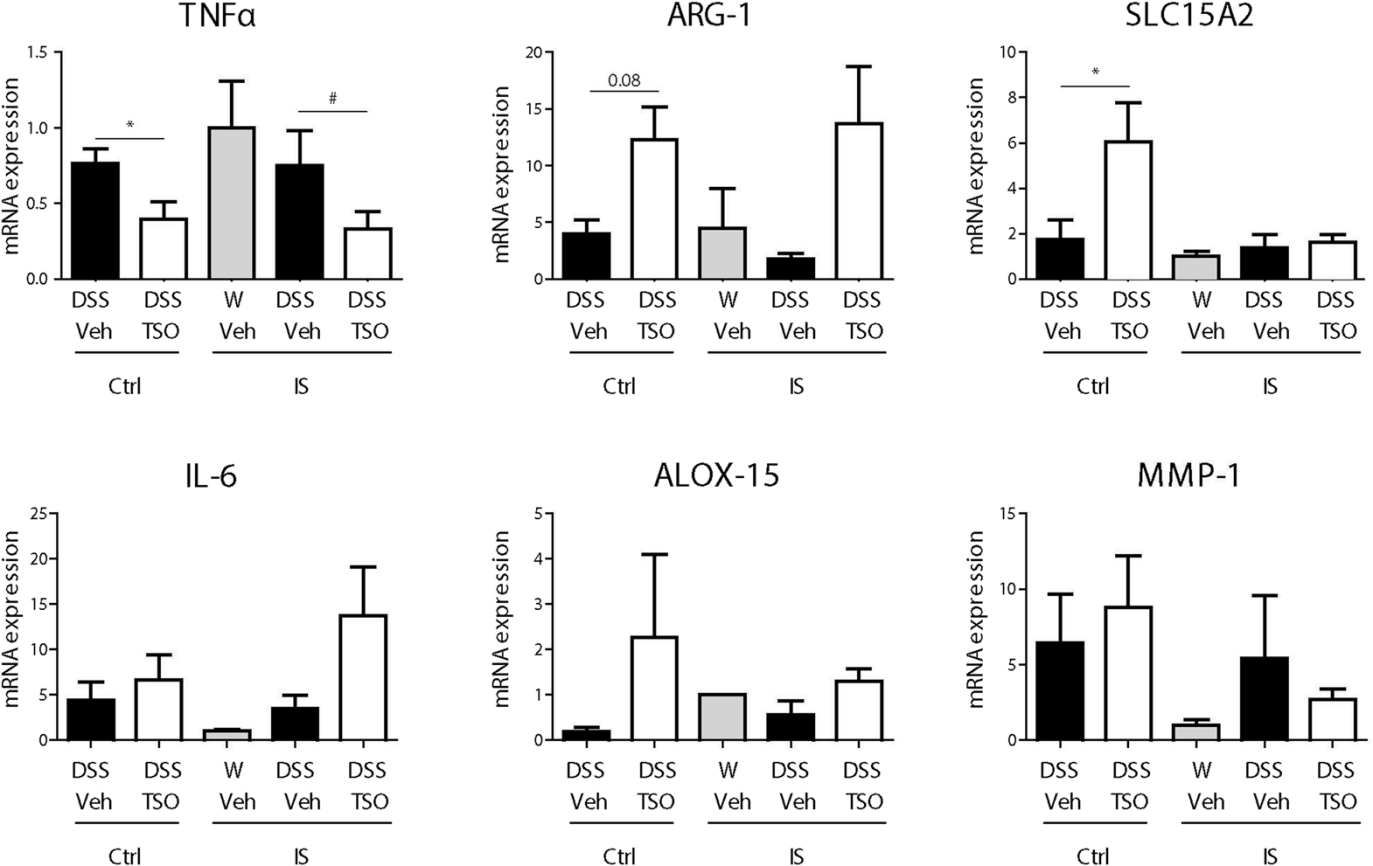
Effects of immunosuppression on the expression of different immune response markers in LPMC. LPMC where isolated from the caecal mucosa of control (ctrl) and immunosuppressed (IS) rabbits. Expression of selected mRNAs was determined by rt-qPCR relative to GAPDH as housekeeping gene. Bars represent Mean ± SEM from two pooled experiments. n = 2–4 animals per group. *P < 0.05, Mann-Whitney test.

Analysis of immune response markers in LPMC showed that immunosuppression reduced the increase in markers of alternative macrophage activation ARG-1 and ALOX-15 (Fig. 9). Similarly, the increase in expression of SLC15A2 was abrogated, whereas mRNA levels of IL-6 increased in IS TSO DSS animals, although not significantly. On the other hand, the reduction in TNFα mRNA levels observed in the immunocompetent rabbits persisted.

## Discussion

The chronic course of inflammatory bowel disease requires a long-term treatment that maximizes the anti-inflammatory action while minimizing the systemic effects. Unfortunately, currently available therapies have limitations in both efficacy and/or tolerability. The first human trials investigating a therapeutic effect of *Trichuris suis* in UC and CD patients were promising^23,29^. Yet, larger multicentre studies could not show a significant effect of *T. suis* ova (TSO) in comparison to placebo in mild to moderate IBD patients. Currently, the available evidence in support of TSO therapy is judged insufficient^20^. Furthermore, the mechanisms underlying TSO therapy at molecular level as well as the safety in immunosuppressed individuals could not be investigated so far due to the lack of a suitable animal model.

Since *T. suis* shows a similar transient colonization in both humans and rabbits, we chose to study the efficacy and safety of TSO in a recently developed rabbit model of colitis^25^.

Consistent with human studies, we found that a preventive treatment with three doses of 2500 TSO protected from colitis-induced weight loss, reduced the disease activity index and limited the caecal pathology.

Intriguingly, whole genome transcriptome analysis of caecal LPMC and IEC samples showed a strong effect of TSO treatment on LPMC gene expression, whereas the effect on IEC was milder. This observation agrees with evidence that *T. suis* excreted or secreted (ES) compounds (such as glycans) increase epithelial permeability, and allows the passage of immunomodulatory soluble compounds to the lamina propria^30,31^. Supporting these findings, we found that several mRNAs involved in the degradation of connective tissues were over-expressed in caecal IEC upon TSO treatment. *T. suis* ES products have been linked to chemokine, T-cell receptor and TGFβ signalling as well as leukocyte trans endothelial migration^31^. Accordingly, we observed a TSO-induced decrease in expression of mRNAs involved in cell-adhesion, endothelium-leukocytes interactions and chemotaxis. Furthermore, TSO also limited the DSS-induced increase in expression of mRNAs involved in Th17 immune response and were associated with an increase in expression of mRNAs involved in innate inflammation, IL-4 and histamine signalling. Whilst these observations largely accord with the typical anti-helminth response, a species-specific response to *T. suis* might occur, especially given the profound differences observed when comparing its transcriptome with those of other well studied helminths^32^. Our data suggest that TSO treatment predominantly affects LP immune cells. Since the majority of IBD patients receive an immunosuppressive therapy^33^, the protective modulation of the immune response could be lost, thus rendering the therapy ineffective. Furthermore, although *T. suis* is not a human parasite, an aberrant migration of *Trichuris* particularly in an immunosuppressed host with impaired gut barrier function cannot be excluded *a priori*. In humans, trichuriasis symptoms can range from mild digestive tract distress to anaemia, dysentery, bleeding, abdominal pain and more generalized effects, such as nausea, vomiting, anaemia and peripheral blood eosinophilia^34^.

Although no side-effects were reported in the published clinical trials in IBD^18,23,29,35^, the disease symptoms might mask the consequences of a *Trichuris* induced intestinal inflammation. In fact, symptoms have been observed in studies testing a TSO therapy for non-gastrointestinal diseases. In a randomized double-blinded placebo-controlled clinical trial in allergic rhinitis, patients ingesting TSO had a 3 to 19-fold higher rate of gastrointestinal episodes (flatulence, diarrhoea and abdominal pain) compared with placebo subjects^36,37^. Similarly, in two small pilot studies in multiple sclerosis, patients experienced mild transient symptoms at about 30 days after the first TSO dose^38,39^.

To address the lack of systematic studies of TSO in immunosuppressed individuals we tested the outcome of TSO administration in immunosuppressed rabbits. To achieve immunosuppression, we used a combination of cyclosporine and methylprednisolone. Cyclosporine acts rapidly and is effective in the management of severe UC, whereas corticosteroids are used for moderate to severe relapses of both IBD forms^40^.

Importantly, we show that in immunosuppressed rabbits, TSO exacerbated colitis and increased the mortality of the affected animals. Our results highlight the dangers of helminth therapy in immunosuppressed hosts. Histology revealed the presence of late-stage larvae in the caecum, suggesting a failure of the anti-helminth response when the intestinal barrier function is damaged and the immune response impaired. Our histological findings resemble those observed in an immunosuppressed CD patient that developed an iatrogenic *Trichuris* infection after treatment with TSO^24^.

Since *Trichuris* species are known to modulate the intestinal microbiota^41,42^ we investigated whether TSO treatment would modulate the bacterial communities and whether these changes could be associated with the observed protective effect. Analysis of microbiota revealed clear differences in the composition of the caecal and faecal microbiota of DSS treated rabbits in comparison to water treated controls.

As is commonly observed in IBD patients in comparison to healthy controls, the faecal microbiota of DSS-treated animals showed a significant reduction in community richness^43^. Importantly, TSO prevented this DSS-induced reduction, although– in contrast to previous reports from *Trichuris* infected individuals^44^ – healthy TSO-infected rabbits had a slight, albeit not significant, reduction in diversity. Our results confirm the trend observed in colitic Macaques after therapeutic treatment with *T. trichiura* ^42^.

Our analysis identified several OTUs whose abundance in the rabbit microbiome correlated strongly with different indicators of colitis severity. The caecal microbiota of DSS treated rabbits prominently included *Spiroplasma*, *Eubacterium* and *Clostridium* OTUs as well as members of the *Bacteroidetes* phylum, in particular *B. dorei*. Increased abundance of *B. dorei* and other *Bacteroides* species has been implicated in inflammation in several immune-related diseases including UC, celiac disease and collagenous colitis^45–47^. TSO treatment significantly reduced the abundance of these taxa, comparable to the effect recently described for *Heligmosoides polygyrus* infection in mice^48^. TSO also prevented the increase of OTUs belonging to the family *Desulfovibrionaceae* that have been implicated in UC pathogenesis via the production of hydrogen sulphide, which might exacerbate mucosal inflammation and ulceration^49^.

In both caecal and faecal samples, OTUs belonging to the *Clostridium* genus increased independently of TSO-treatment. Similarly, selected *Clostridium* species were strongly increased in treatment naive CD patients^50^. In contrast, other *Clostridiales* order belonging in particular to the genus *Ruminococcus* were strongly reduced in DSS-treated animals; a trend also observed in CD^50^ and UC^51^ patients.

To our knowledge, no study investigating the effect of immune suppression on the intestinal microbiota has been published. Interestingly, the DSS-induced reduction in alpha diversity was lost in immunosuppressed animals, suggesting that immune-suppression can mitigate colitis-associated dysbiosis. Immunosuppressed rabbits had a reduced frequency of OTUs positively associated with colitis markers and an increased frequency of several OTUs that had a negative correlation with colitis severity. This is in accordance with the anti-inflammatory effect exerted by the immunosuppressive treatment.

Treatment with TSO abrogated the reduction of the majority of these colitis-associated species in the IS DSS group. At the same time, TSO prevented the increase of *Clostridiales* associated with reduced colitis severity, including members of *Eubacteriaceae*, *Corynebacterium* and *Ruminococcaceae* ^52^. Recent studies showed that commensal *Clostridium* bacteria promote anti-inflammatory immune responses by expanding and activating regulatory T cells^53^. A reduction of their protective regulatory stimuli might contribute to the exacerbated inflammation observed in the immunosuppressed TSO group. Whether the observed changes are causal to the increased colitis severity or rather, a consequence of the disturbed environment warrants further investigation.

In summary, our study provides further evidence for the therapeutic efficacy of a preventive TSO therapy in intestinal inflammation. Yet, immunosuppression interferes with TSO treatment, counteracts its therapeutic effect and might predispose towards adverse effects. Based on these findings, caution should be exercised when treating immunosuppressed IBD patients with TSO or other therapeutic parasites. Investigations into the therapeutic effect of parasite-derived products might allow exploiting the therapeutic potential of helminths without the risks associated with a therapeutic helminth infection.

## Materials and Methods

### Rabbits

All animal experiments were carried out according to Swiss animal welfare laws and approved by the veterinary office of Zurich (licenses No. 128-2010 and 231-3013). New Zealand white rabbits (Charles River, Kisslegg, Germany) weighing 1.9–2.1 kg (8–10 weeks of age) were maintained single-housed with water and food (standard rabbit maintenance diet – Provimi Kliba AG, Kaiseraugst, Switzerland, hay and straw) *ad libitum* on a 12:12 hour light/dark cycle. Upon arrival, animals were kept for at least 4 days under routine husbandry. Afterwards, drinking water was substituted by organic fennel tea (Hipp, Pfaffenhofen, Germany) *ad libitum* as fennel tea can mask the bitter taste of DSS that would reduce liquid intake in rabbits.

### TSO administration

Rabbits were randomly assigned to treatment groups receiving either suspensions of 2500 TSO (embryonated *T. suis* eggs, supplied as ready-to-use inoculation doses) or the vehicle (Phosphoric acid buffer pH 5.0 with 0.05% potassium sorbate) in three oral doses at day 1, day 14 and day 21. Animals were fasted overnight prior to TSO administration and sedated with 0.4 ml Hypnorm (VetaPharma, Leeds, UK), *s.c*. The TSO suspension or vehicle (volume 15 ml) was completely filled in a syringe and administered to the animal using a gastric tube (i.e. Ruesch Katheter, CH14, REF 402101, Kernen, Germany). The animals were fed shortly after administration.

### Colitis induction and clinical evaluation

DSS Colitis was induced at day 26 as described previously^25^. Rabbits received DSS (MP Biomedicals, Illkirch, France) dissolved in cold fennel tea at 0.1% w/v for 5 days. Control rabbits received cold fennel tea only. Animal weight, food and beverage intake, stool appearance and behaviour were monitored daily. A DAI was calculated according to Supplementary Table 2.

### Immunosuppression

Immunosuppressive treatment was started 2 weeks prior to the first TSO gavage. The rabbits received cyclosporine (100 μl/kg/day, Sandimmun Neoral Trink Lösung 100 mg/ml; Novartis Pharma Schweiz AG, Basel, Switzerland) and methylprednisolone (1 mg/kg/day, 6α-Methylprednisolone 21-hemisuccinate sodium salt -lyophilized powder, Sigma-Aldrich, Munich, Germany) for 2 weeks daily by oral administration. Afterwards, the dose was reduced to half and the treatment continued until the end of the experiment. Control rabbits received vehicle (12% V/V EtOH). 5 ml of blood were collected from the marginal ear vein prior to immunosuppressive treatment and at day 1, 14, 21, and 35 and sent to the clinic of haematology (USZ, Zurich, Switzerland) for a complete blood count.

### Euthanasia and Organ sampling

Euthanasia was performed on day 35 (unless described otherwise) with 150 mg/kg Pentobarbital i.v. in deeply sedated animals (85 mg/kg ketamine hydrochloride, Vétoquinol, Bern, Switzerland and 4 mg/kg xylazine, Bayer, Lyssach, Switzerland).

The abdominal cavity was exposed by a midline laparotomy and samples were collected from the ileum, jejunum, duodenum, caecum and colon. For RNA extraction and myeloperoxidase activity analysis, the excised samples (0.5 cm in length) were opened by a longitudinal incision and rinsed with cold PBS. One cm^2^ sections of the caecum were extensively washed with cold PBS until complete removal of the luminal content. The samples were immediately snap-frozen in liquid nitrogen and stored at −80 °C until analysis. For histology, samples (three 0.5 cm^2^ sections from different regions of the caecum or one 0.5 cm long section of other intestine segments) were collected. The samples were carefully washed and fixed with phosphate buffered 10% formalin solution. For RNA isolation, caecal samples (2 cm^2^) were extensively washed with cold PBS and stored on ice in 5% BSA in PBS until further processing.

### Isolation of caecal LPMC and IEC

The dissected specimens were washed with Ca^+^- and Mg^+^-free PBS, the caecal fold was removed and discarded. The tissue was cut and incubated in medium containing 20 mM EDTA (Sigma-Aldrich) for 30 min at 37 °C on a shaking platform (150 rpm). IECs were detached by vortexing and passing through a 70 µm cell strainer (BD Biosciences, Erembodegem, Belgium). The IEC were washed twice, pelleted, resuspended in RLT buffer (Qiagen, Hilden, Germany), snap-frozen in liquid nitrogen and stored at −80 °C for later analysis. The remaining tissue containing LP with muscle layer was collected and incubated in one μg/ml collagenase type I CLS (Worthington Biochemical Corp., Freehold, New Jersey, USA) at 37 °C on a shaking platform (300 rpm). After 15 minutes incubation, the suspension was vortexed and filtered through a 70 µl strainer. Cells were resuspended in 5% BSA in PBS. The undigested tissue was incubated with fresh collagenase solution for additional 15 minutes. The digestion was repeated three times and the washed LPMC were pooled and resuspended in DMEM with 5% FCS. LPMC were purified using Ficoll-Paque PLUS (GE Healthcare Europe GmbH, Freiburg Germany) gradient centrifugation for 40 min at 1200 rpm. The viability of the cells was confirmed by trypan blue staining. Cells were resuspended in RLT buffer (Qiagen, Hilden, Germany), snap-frozen in liquid nitrogen and stored at −80 °C for later analysis.

### RNA isolation and genome wide mRNA expression analysis

Total RNA was isolated with the Qiacube system using the RNeasy Mini Kit with DNase digestion (Qiagen, Hilden, Germany) to eliminate genomic DNA. RNA integrity and quantity was determined on the Agilent 2100 Bioanalyzer (Agilent; Palo Alto, CA, USA). Samples with an integrity score ≥6.8 were sent to the Functional Genomics Centre Zurich (FGCZ) for sequencing.

Sequencing of the 2 × 250 bp inserts was performed on the Illumina HiSeq. 2500 v4 platform at the FGCZ. Fold change (FC) was used to express the changes in average gene expression between studied groups. FC was normalized against the control group. The following cut-offs were applied to identify differentially expressed genes: *FDR*-*corrected-p-Value* ≤ 0.05 and log2 (FC) ≥ |1.0|. The RNAseq data have been deposited in NCBI’s Gene Expression Omnibus and are accessible through GEO Series accession number GSE77372.

The ENSEMBL IDs were annotated using BetterBunny augmented annotation and analysis of rabbit genes (http://cptweb.cpt.wayne.edu)^54^. MetaCore™ (Thomson Reuters, http://portal.genego.com) was used to perform network analyses. Process networks (PN, groups of genes involved in main signalling and metabolic processes in the cell in the MetaCore™ database) were considered significant with a p-Value ≤ 0.05 and were listed on the basis of their relevance to IBD pathology.

### Histopathological evaluation of colitis

After careful dissection and fixation, tissues were embedded in paraffin. Serial sections of five μm were cut using a microtome (Carl Zeiss AG, Feldbach, Switzerland) and stained with haematoxylin-eosin (HE). The histological changes in the caecum were quantified in a blinded manner by two investigators (range 1–24) for morphological features (villous stunting, villous epithelial injury and crypt distortion) and infiltration of immune cells (intraepithelial lymphocytes and infiltrating lymphocytes and plasma cells in the LP) as outlined in Supplementary Table 3.

### RNA extraction and quantitative real-time PCR

Total RNA was isolated using the RNeasy Mini Kit (Qiagen, Hilden, Germany) following the manufacturer’s recommendations. cDNA was synthesized with the High-Capacity cDNA Reverse Transcription Kit (Life Technologies, Carlsbad, California, U.S.A). Gene expression was determined with a *Taq*Man® Gene Expression Assay (#Oc04097051_m1 IL-6; #Oc04250656_m1 MMP1; #Oc03398293_m1 PTGS2 (COX2); #Oc03398448_m1 SLC15A2; #Oc03397715_m1 TNFα; Oc03823548_s1 ALOX-15A2; Oc03397217_m1 ARG-1; Applied Biosystems). Glyceraldehyde 3-phosphate dehydrogenase (GAPDH) gene expression was measured as endogenous control (#Oc03823402_g1, Applied Biosystems) and used for calculation of relative mRNA expression by the ΔΔCt method. All samples were analysed in triplicate.

### Faecal and luminal microbiota analysis

Fresh faecal samples were collected at the start of the experiment and at termination. Luminal contents were collected from the caecum after euthanasia. Samples were snap-frozen in liquid nitrogen and stored at −80 °C for later analysis. DNA isolation was performed using the PowerLyzer PowerSoil Kit (MO BIO Laboratories, Carlsbad, CA USA) using 0.25 g faeces or luminal content according to the manufacturer’s protocol. Approximate yield (ng/ml) was first determined by spectrophotometry using the NanoDrop 2000 (Thermo Fisher Scientific, Waltham, MA, USA); samples with DNA concentrations >10 ng/ml were selected for subsequent analyses.

Targeted sequencing of the 16S rRNA gene was performed at Microsynth (Balgach, Switzerland). The V4 region was amplified with primers described by Caporaso *et al*.^55^ and barcoded in a twostep PCR approach. Paired-end sequencing was performed on an Illumina MiSeq system. Pre-filtered paired reads were demultiplexed and stitched following mothur’s MiSeq SOP (http://www.mothur.org/wiki/MiSeq_SOP, accessed May 2015^56,57^) and filtered for length and putative chimeric PCR products (UCHIME^58^). Reads were taxonomically classified and mapped to reference Operational Taxonomic Units (OTUs) at different levels of sequence similarity using *MAPseq* ^59^ against an average linkage pre-clustered reference database of full length 16S rDNA sequences. Unmapped sequences were aligned to a bacterial reference 16S model as provided in the package *ssu-align* (http://selab.janelia.org/software/ssu-align/ ref.^60^) using the structure-aware aligner *Infernal* ^61^. After removing sequences which did not align to the model or were not classified as bacterial by MAPseq, reads were pre-clustered (one mismatch abundance-sorted single linkage) and then hierarchically clustered into OTUs using *hpc-clust* ^62^ according to the average linkage algorithm which has previously been shown to provide reproducible and consistent clustering^63^. OTU sets were generated at different levels of sequence similarity; in this manuscript, data are shown for OTUs at 98% sequence similarity. Further biological analyses were performed using R scripts, particularly relying on the R packages *vegan* ^27^, *phyloseq* ^64^ and *edgeR* ^65^. Raw sequence data has been deposited in the NCBI Sequence Read Archive (BioProject ID PRJNA309382, http://www.ncbi.nlm.nih.gov/bioproject/PRJNA309382). Analysis scripts are available via http://github.com/defleury/2016_Leonardi_et_al_rabbit_tso. Data can be accessed at http://meringlab.org/suppdata/2016-rabbit_tso/.

### Trichuris suis detection by PCR

Tissue samples (colon, ileum, caecum, brain, kidney, spleen) were collected after euthanasia and immediately snap-frozen in N_2_. Samples were sent to IBR Inc (Matzingen, Switzerland) for detection by PCR. Primers for the ITS2 gene were used (Supplementary Table 4). Primers are based on the *T.suis* specific internal transcribed spacers (ITS)1-5.8S-ITS2 segment of the ribosomal DNA^66^. The ITS2 copy number is proportional to the number of *T. suis* larvae.

### Analysis of peroxidase activity

Peroxidase activity in caecal lysates was measured as previously described^25^. To achieve specificity for neutrophil derived MPO, the assay was conducted in presence of the EPO inhibitor aminotriazole (3-Amino-1H-1,2,4-triazole, 95%; Brunschwig, Basel, Switzerland) at pH 6.0^67^. MPO activity (indicated as arbitrary units U/g.s) was calculated as mean absorbance (460 nm) per incubation time per protein content of the sample in grams determined by BCA assay (ThermoFisher, Rockford, USA).

### Faecal flotation

5 g of fresh faeces were collected 1 day before and 1 day after each TSO-gavage and 9 days after start of the DSS-treatment. The samples were suspended immediately in 300 ml water, filtered through a 1 mm sieve and incubated at RT for 30 min. The supernatant was discarded and 1 ml of the sediment was resuspended in 10 ml Sheather’s solution (454 g Sucrose in 355 ml water with 6 ml 37% formalin), mixed briefly and centrifuged 5 min at 500 g. 4 drops were collected with a smear loop, transferred to a slide and examined at 10x lens objective, (changing the plane of focus during the examination). As a positive control, 1 ml of the TSO-gavage solution was suspended in 10 ml Sheather’s solution and analysed as described above.

### Statistical analysis

The data obtained from this study were analysed using GraphPad Prism (version 5.04) and the R statistical framework (version 3.1.2). If not otherwise indicated, Mann - Whitney *U*-test for two independent samples was used for the comparison of the treatment groups, data are shown as mean ± SEM.

## Electronic supplementary material

Supplementary Figures and Tables

OTU association tables

OTU correlation tables
