# Supplementary material for: Preventive Trichuris suis ova (TSO) treatment protects immunocompetent rabbits from DSS colitis but may be detrimental under conditions of immunosuppression
Source: Sci Rep. 2017 Nov 28;7:16500. doi: 10.1038/s41598-017-16287-4 (PMC5705695; doi:10.1038/s41598-017-16287-4)
Supplement: Supplementary file 1 — Supplementary Figures and Tables [file 41598_2017_16287_MOESM1_ESM.pdf]

Preventive *Trichuris suis* ova (TSO) treatment protects immunocompetent rabbits from DSS colitis but may be detrimental under conditions of immunosuppression

Irina Leonardi[1†], Alexandra Gerstgrasser[1], Thomas SB Schmidt[2], Flora Nicholls[3], Bernhard Tewes[4], Roland Greinwald[4], Christian von Mering[2], Gerhard Rogler[1], Isabelle Frey-Wagner[1*]

Supplementary figures

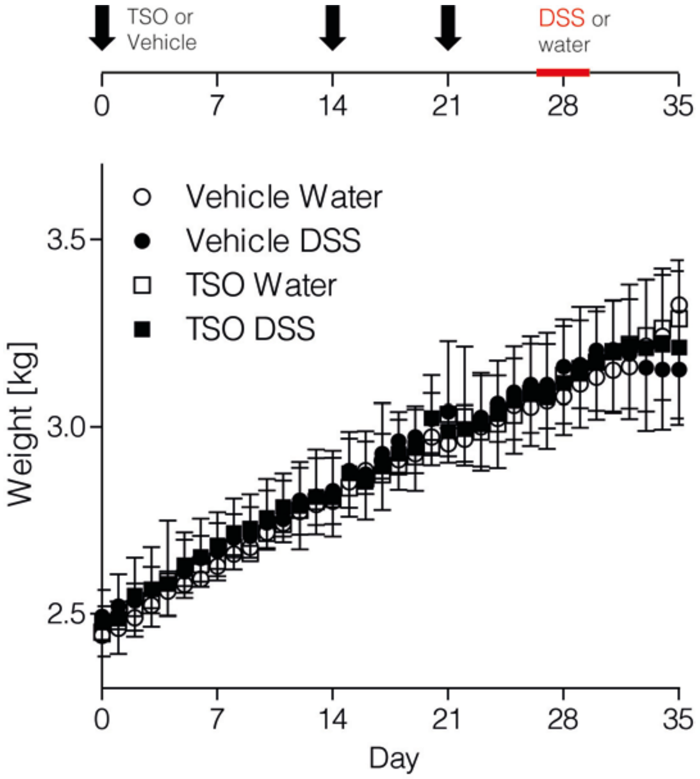

Supplementary Figure 1: Preventive treatment with TSO in a model of DSS induced acute colitis Weight progression from day 0 (first TSO/Vehicle treatment) to day 35 (day of euthanasia and organ sampling). Colitis was induced at day 26 by administration of 0.1 % DSS (w/v) in the daily beverage for 5 days. Dots represent mean ± SD.

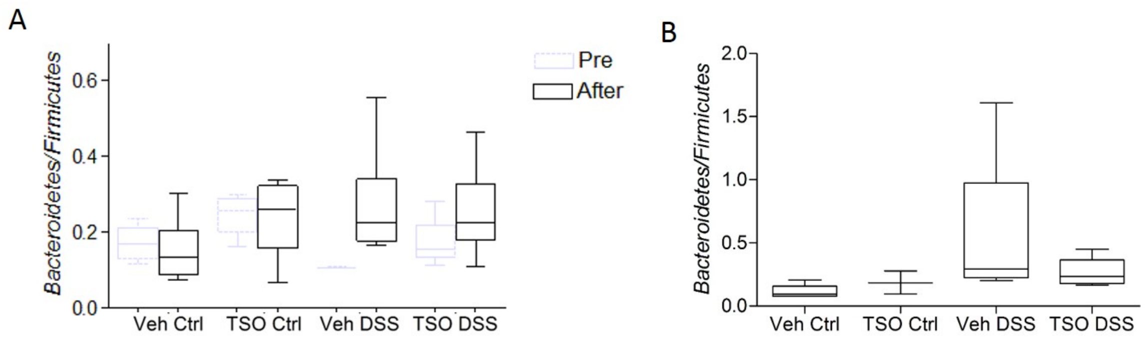

Supplementary Figure 2: Bacteroidetes to Firmicutes ratio in faeces (A) and cecal contents (B)

A permutational multivariate ANOVA (PERMANOVA). Cecum: Drug P=0.05, Parasite*Drug P=9.076; Feces: Drug P=0.043. The abundance of Bacteroidetes and Firmicutes was obtained by Taxonomy analysis of the 16S Data at phylum level.

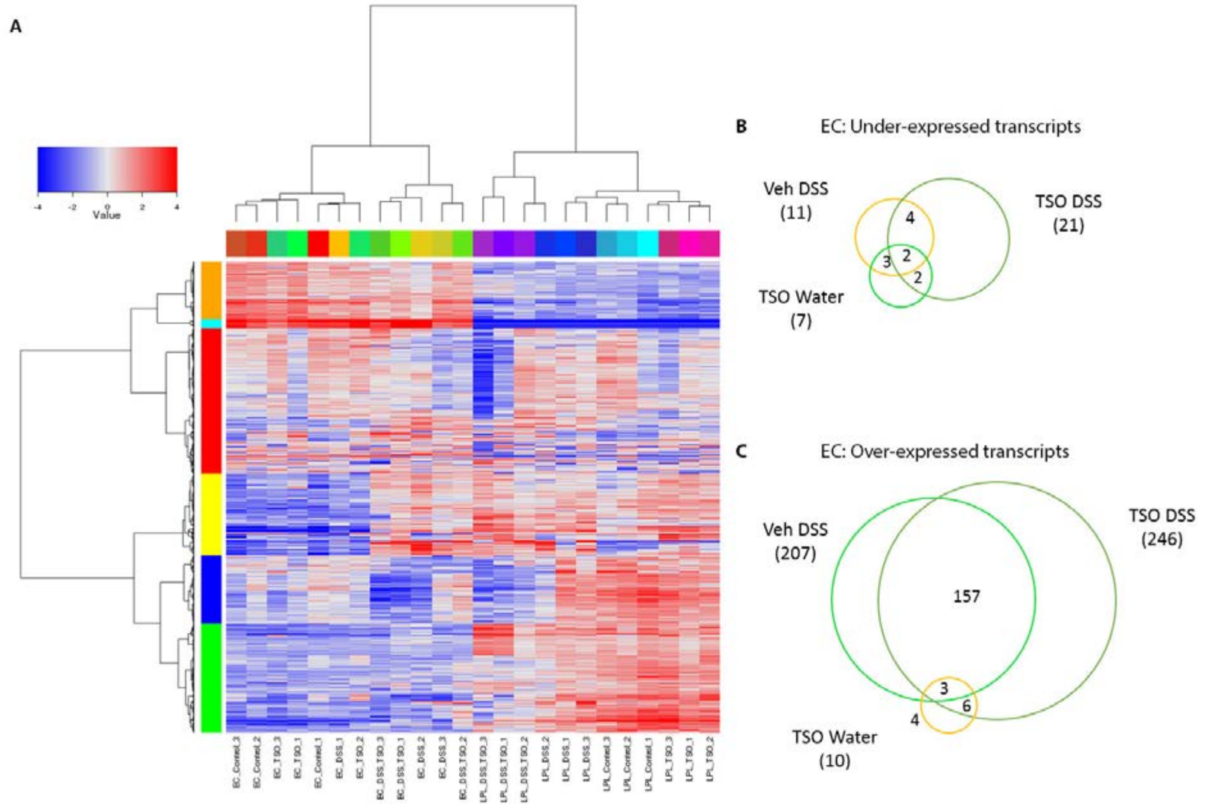

Supplementary Figure 3: Genomic signature of differentially expressed transcripts in EC and LPMC. RNA was prepared from cells isolated from rabbit ceca (n=3 per group) and subjected to genome wide expression analysis. Hierarchical cluster analysis was used to sort expression according to treatment and cell type. (A) Heatmap of altered expression according to treatment and cell type. Each column displays the genomic signature for 1 rabbit. Under-expressed (B) and over-expressed (C) transcripts in EC isolated form TSO DSS, Veh DSS and TSO Water rabbits in comparison to the gene expression in control Veh Water rabbits. Transcripts having a fold change log2|FC|>1 and $P < 0.05$ were considered differentially expressed genes and were included in the analysis.

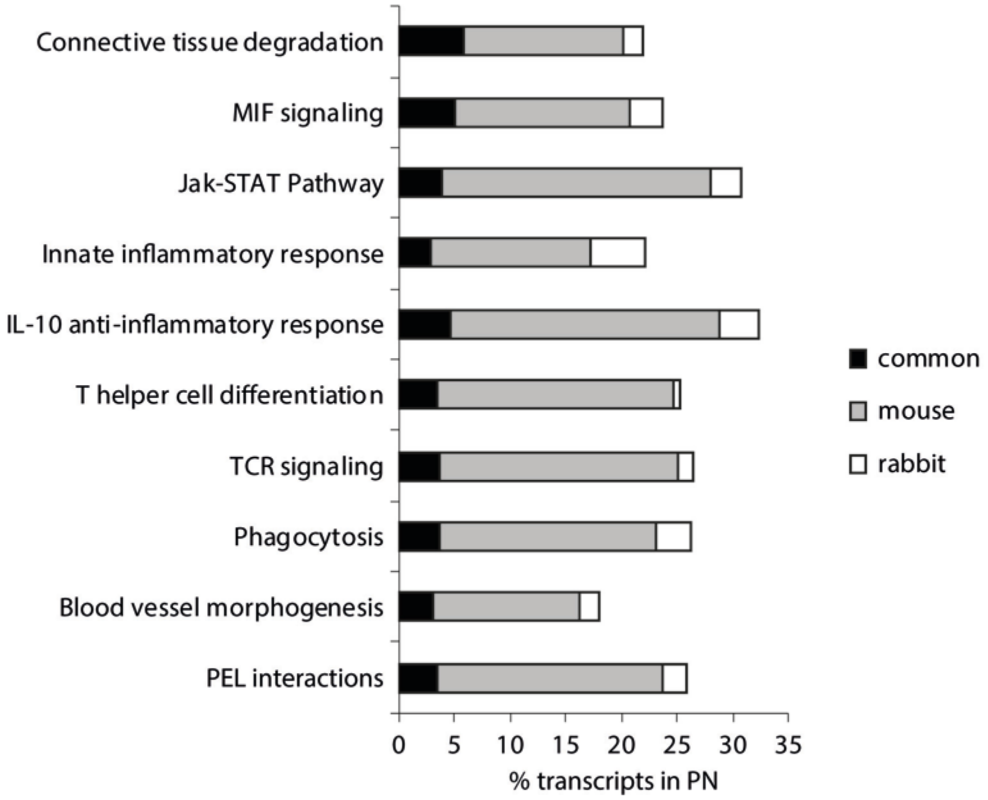

Supplementary Figure 4: Process network (PN) analysis of transcripts differentially expressed in LPMC and IEC from *T. suis* infected rabbits and in the caecal tissues of *T. muris* infected mice. Black bars show the percentage of transcripts affected in both rabbit and mice. The percentage of unique transcripts is shown in white bars for rabbit and in grey bars for mouse. MIF: Macrophage migration inhibitory factor, PEL: Platelet-endothelium-leucocyte interactions, TCR: T cell receptor. Analysis was performed with MetaCore. Genes with FDR < 0.1 where included in the analysis.

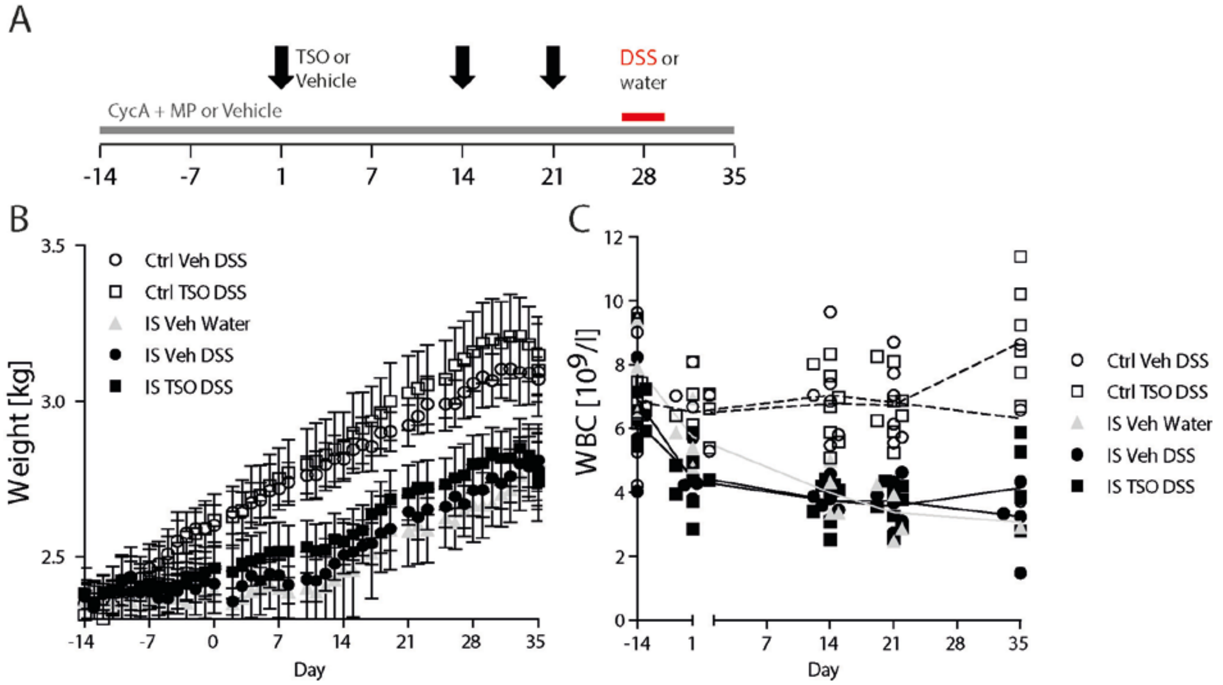

Supplementary Figure 5: Preventive treatment with TSO in a model of DSS induced acute colitis in immunosuppressed (IS) and immunocompetent (Ctrl) rabbits.

A. Experimental layout: IS rabbits received Cyclosporine A (CycA) and Methylprednisolone (MP) daily. B. Weight progression from day -14 (start of IS treatment) to day 35 (day of euthanasia and organ sampling) Data show mean ± SD from one representative experiment. C. total WBC was determined prior to the start of the experiment (d0) and at day 1, 14, 21 and 35. Dots represent single animals, lines connect arithmetic mean. Data are pooled from 3 independent experiments.

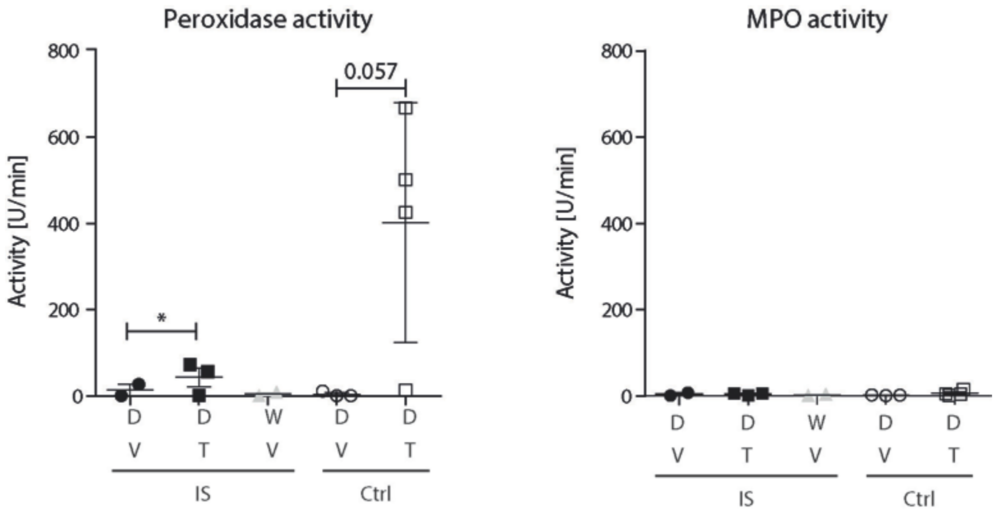

Supplementary Figure 6: Neutrophil and eosinophil infiltration in immunosuppressed rabbits. Infiltration into the caecum was determined indirectly by measuring the caecal peroxidase activity. Caecal specimens were excised and homogenized. The supernatants were assayed for peroxidase activity with or without the selective eosinophil-peroxidase inhibitor aminotriazole (AMT); activity was normalized to the total protein content as determined by BCA test. D: DSS, W: water, V: Vehicle, T: TSO, IS: immunosuppressed, Ctrl: control. Dots represent single animals, bars show mean ± SEM, *P < 0.05, two-sided p-Value, unpaired t-test

Supplementary Tables

Supplementary Table 1: Overexpressed genes in LPMC and IEC from TSO infected rabbits and in the caecal tissues of *T. muris* infected mice.

Functional annotation was performed using the David Functional Annotation Tool, 6.7, NIAID/NIH. Genes with FDR < 0.1 where included in the analysis. Mouse caecum expression data where obtained from Foth, 2014[44].

Gene ID	KEGG pathway	Biocarta	Gene name
**adam8**			a disintegrin and metallopeptidase domain 8
**alox15**	Arachidonic acid metabolism,		arachidonate 15-lipoxygenase
**arg1**	Arginine and proline metabolism,	Catabolic Pathways for Arginine , Histidine, Glutamate, Glutamine and Proline,	arginase, liver
**arhgdib**	Neurotrophin signalling pathway,	Caspase Cascade in Apoptosis, D4-GDI Signalling Pathway, FAS signalling pathway (CD95), HIV-I Nef, TNFR1 Signalling Pathway	Rho, GDP dissociation inhibitor (GDI) beta
**arid5a**			AT rich interactive domain 5A (MRF1-like)
**basp1**			brain abundant, membrane attached signal protein 1
**c1qb**	Complement and coagulation cascades, Prion diseases, Systemic lupus erythematosus,		complement component 1, q subcomponent, C chain
**c1qc**	Complement and coagulation cascades, Prion diseases, Systemic lupus erythematosus,		complement component 1, q subcomponent, beta polypeptide
**ccrl2**			chemokine (C-C motif) receptor-like 2
**cd14**	MAPK signalling pathway, Toll-like receptor signalling pathway, Hematopoietic cell lineage, Regulation of actin cytoskeleton,	Inactivation of Gsk3 by AKT causes accumulation of b-catenin in Alveolar Macrophages,	CD14 antigen
**cfp**		Alternative Complement Pathway,	complement factor properdin
**chi3l1**			chitinase 3-like 1
**ciita**	Antigen processing and presentation, Primary immunodeficiency,		class II transactivator

**csf1r**	Cytokine-cytokine receptor interaction, Endocytosis, Hematopoietic cell lineage, Pathways in cancer,	CBL mediated ligand-induced downregulation of EGF receptors, METS effect on Macrophage Differentiation,	colony stimulating factor 1 receptor
**csf2rb**	Cytokine-cytokine receptor interaction, Apoptosis, Jak-STAT signalling pathway,	IL 3 signalling pathway,	colony stimulating factor 2 receptor, beta, low-affinity (granulocyte-macrophage)
**dhrs9**	Retinol metabolism,		dehydrogenase/reductase (SDR family) member 9
**fgr**	Chemokine signalling pathway,	Roles of αβγ -arrestin-dependent Recruitment of Src Kinases in GPCR Signalling,	Gardner-Rasheed feline sarcoma viral (Fgr) oncogene homolog
**gpr141**			G protein-coupled receptor 141
**hck**	Chemokine signalling pathway, Fc gamma R-mediated phagocytosis,	Roles of αβγ-arrestin-dependent Recruitment of Src Kinases in GPCR Signalling,	hemopoietic cell kinase
**hdc**	Histidine metabolism,		histidine decarboxylase
**hk3**	Glycolysis / Gluconeogenesis, Fructose and mannose metabolism, Galactose metabolism, Starch and sucrose metabolism, Amino sugar and nucleotide sugar metabolism, Insulin signalling pathway, Type II diabetes mellitus,		hexokinase 3
**il1b**	MAPK signalling pathway, Cytokine-cytokine receptor interaction, Apoptosis, Toll-like receptor signalling pathway, NOD-like receptor signalling pathway, Cytosolic DNA-sensing pathway, Hematopoietic cell lineage, Type I diabetes mellitus, Alzheimer's disease, Prion diseases, Graft-versus-host disease,	Signal transduction through IL1R, IL 5 Signalling Pathway, Msp/Ron Receptor Signalling Pathway, NFkB activation by Nontypeable Hemophilus influenzae,	interleukin 1 beta
**il1r2**	MAPK signalling pathway, Cytokine-cytokine receptor interaction, Hematopoietic cell lineage,		interleukin 1 receptor, type II
**il1rl1**			interleukin 1 receptor-like 1

**il6**	Cytokine-cytokine receptor interaction, Toll-like receptor signalling pathway, NOD-like receptor signalling pathway, Cytosolic DNA-sensing pathway, Jak-STAT signalling pathway, Hematopoietic cell lineage, Intestinal immune network for IgA production, Prion diseases, Pathways in cancer, Graft-versus-host disease, Hypertrophic cardiomyopathy (HCM),	Cytokine Network, Erythrocyte Differentiation Pathway, Role of ERBB2 in Signal Transduction and Oncology, IL-10 Anti-inflammatory Signalling Pathway, IL 17 Signalling Pathway, Signal transduction through IL1R, IL 5 Signalling Pathway, IL 6 signalling pathway, Cytokines and Inflammatory Response, Regulation of hematopoiesis by cytokines,	interleukin 6
**irf5**	Toll-like receptor signalling pathway,		interferon regulatory factor 5
**itgb7**	Focal adhesion, ECM-receptor interaction, Cell adhesion molecules (CAMs), Intestinal immune network for IgA production, Regulation of actin cytoskeleton, Hypertrophic cardiomyopathy (HCM), Arrhythmogenic right ventricular cardiomyopathy (ARVC), Dilated cardiomyopathy,		integrin beta 7
**itk**	Chemokine signalling pathway, T cell receptor signalling pathway, Leukocyte transendothelial migration,	The Co-Stimulatory Signal During T-cell Activation,	IL2-inducible T-cell kinase
**lcp2**	Natural killer cell mediated cytotoxicity, T cell receptor signalling pathway, Fc epsilon RI signalling pathway,		lymphocyte cytosolic protein 2
**lpcat2**			lysophosphatidylcholine acyltransferase 2
**lrrc33**			leucine rich repeat containing 33
**plek**			pleckstrin
**ptafr**	Calcium signalling pathway, Neuroactive ligand-receptor interaction,		platelet-activating factor receptor

**ptpn7**	MAPK signalling pathway,		protein tyrosine phosphatase, non-receptor type 7
**sell**	Cell adhesion molecules (CAMs),	Adhesion Molecules on Lymphocyte, Monocyte and its Surface Molecules, Neutrophil and Its Surface Molecules,	selectin, lymphocyte
**serpinb2**		Fibrinolysis Pathway,	serine (or cysteine) peptidase inhibitor, clade B, member 2
**sh3kbp1**	Endocytosis,	CBL mediated ligand-induced downregulation of EGF receptors,	SH3-domain kinase binding protein 1
**sla**			src-like adaptor
**slc45a3**			solute carrier family 45, member 3
**srgn**			serglycin
**tgm1**			transglutaminase 1, K polypeptide
**tnf**	MAPK signalling pathway, Cytokine-cytokine receptor interaction, Apoptosis, TGF-beta signalling pathway, TLR signalling pathway, NLR signalling pathway, RIG-I-like receptor signalling pathway, Hematopoietic cell lineage, ...	Cadmium induces DNA synthesis and proliferation in macrophages, Cytokine Network, Free Radical Induced Apoptosis, HIV-I Nef, …	tumor necrosis factor

Supplementary Table 2: Scoring system for the daily monitoring of the disease activity index.

Score	weight loss	stool appearance and caecotrophs	reduction in food intake	reduction in beverage intake	fur appearance
**0**	None	well-formed solid pellets, 0 caecotrophs	none	none	clean, bright fur
**1**	0%-2%	easy to smear and loose stool, ≤1 caecotrophs	0%-30%	0%-30%	dim fur
**2**	2%-5%	loose stool, 2-3 caecotrophs	30%-60%	30%-60%	shagged fur
**3**	5%-10%	loose smeared stool in cage, 4-5 caecotrophs	60%-90%	60%-90%	smudgy, unclean fur
**4**	> 10%	loose smeared stool in cage, > 5 caecotrophs	> 90%	> 90%	smudgy, stool-stains, smeared anus

Supplementary Table 3: Scoring system for DSS-induced histological changes in the caecum.

	Intestinal architecture			Inflammatory cell infiltrate	
	**Villous stunting**	**Villous epithelial injury**	**Crypt distortion**	**Intraepithelial lymphocytes**	**LP lymphocytes and plasma cells**
**1**	Normal mucosa	Normal mucosa	Normal mucosa	5–10/50 IEL/epithelial cells	25% of the villous lamina propria
**2**	Mild villous stunting	Mild villous epithelial injury	Mild crypt distension, hyperplasia and distortion	11–30 IEL/50 epithelial cells.	25%–50% of the villous lamina propria
**3**	Moderate villous stunting	Moderate villous epithelial injury	Moderate crypt distension, hyperplasia and distortion.	31–50 IEL/ 50 epithelial cells may be focally clustered.	50%–75% of the villous lamina propria.
**4**	Marked villous stunting	Marked villous epithelial injury	Marked crypt distension, hyperplasia and distortion	51–100 IEL/ 50 epithelial cells, may be clustered and at all levels of the epithelium	75% – 100% of the villous lamina propria.

Supplementary Table 4: Primers and standards used for the detection of *T. suis*

Primer	***Sequence***
**Forward primer** ITS2-FW1	5' - CTGCGGAGAGCGGCTAACT – 3'
**Reverse primer** ITS2-RW1	5' - ATGTAGCGACGACGTAGCCAACT – 3'
**Internal Standard Probe** IntStd-TS-P	5' - VIC – TGAAAATGCCAAAGTGACAAG – 3'
Trichuris suis **Probe** ITS-P1	5' - FAM – CAGTACGGAAGCTGCC – 3'
